# A sensory signal related to left-right symmetry modulates intra- and interlimb cutaneous reflexes during locomotion in intact cats

**DOI:** 10.3389/fnsys.2023.1199079

**Published:** 2023-06-09

**Authors:** Stephen Mari, Charly G. Lecomte, Angèle N. Merlet, Johannie Audet, Jonathan Harnie, Ilya A. Rybak, Boris I. Prilutsky, Alain Frigon

**Affiliations:** ^1^Department of Pharmacology-Physiology, Faculty of Medicine and Health Sciences, Université de Sherbrooke, Sherbrooke, QC, Canada; ^2^Department of Neurobiology and Anatomy, Drexel University College of Medicine, Philadelphia, PA, United States; ^3^School of Biological Sciences, Georgia Institute of Technology, Atlanta, GA, United States

**Keywords:** cutaneous reflexes, interlimb coordination, locomotion, modulation, split-belt treadmill

## Abstract

**Introduction:**

During locomotion, cutaneous reflexes play an essential role in rapidly responding to an external perturbation, for example, to prevent a fall when the foot contacts an obstacle. In cats and humans, cutaneous reflexes involve all four limbs and are task- and phase modulated to generate functionally appropriate whole-body responses.

**Methods:**

To assess task-dependent modulation of cutaneous interlimb reflexes, we electrically stimulated the superficial radial or superficial peroneal nerves in adult cats and recorded muscle activity in the four limbs during tied-belt (equal left-right speeds) and split-belt (different left-right speeds) locomotion.

**Results:**

We show that the pattern of intra- and interlimb cutaneous reflexes in fore- and hindlimbs muscles and their phase-dependent modulation were conserved during tied-belt and split-belt locomotion. Short-latency cutaneous reflex responses to muscles of the stimulated limb were more likely to be evoked and phase-modulated when compared to muscles in the other limbs. In some muscles, the degree of reflex modulation was significantly reduced during split-belt locomotion compared to tied-belt conditions. Split-belt locomotion increased the step-by-step variability of left-right symmetry, particularly spatially.

**Discussion:**

These results suggest that sensory signals related to left-right symmetry reduce cutaneous reflex modulation, potentially to avoid destabilizing an unstable pattern.

## 1. Introduction

During locomotion, somatosensory feedback from the moving limbs informs the central nervous system about the body’s posture and its interactions with the external environment (reviewed in Frigon et al., 2021). For instance, when the foot dorsum makes contact with an obstacle during the swing phase, cutaneous inputs allow the central neural controller to rapidly adjust the locomotor pattern to prevent a fall. This reflex response termed the stumbling corrective reaction modifies the contacted leg’s trajectory while reinforcing contralateral leg support, as shown in intact and spinal-transected cats (Forssberg et al., 1977; Prochazka et al., 1978; Forssberg, 1979; Duysens and Loeb, 1980; Wand et al., 1980; Quevedo et al., 2005a) and healthy humans (Schillings et al., 1996; Van Wezel et al., 1997; Zehr et al., 1997). Stimulating the superficial peroneal nerve (SP) innervating the foot dorsum evokes the specific pattern of muscle activations characterizing the stumbling corrective reaction (Quevedo et al., 2005a,b). The same response pattern is found in the forelimbs of intact or decerebrate cats during quadrupedal locomotion after stimulating the superficial radial nerve (SR), which innervates the forepaw dorsum (Matsukawa et al., 1982; Drew and Rossignol, 1985, 1987; Seki and Yamaguchi, 1997; Hurteau et al., 2018). The same stimulus applied to these nerves during stance on the other hand prevents limb flexion, generating a functional response termed the stumbling preventive reaction (Forssberg, 1979; Buford and Smith, 1993; Quevedo et al., 2005a).

Studies in humans have shown the potential role of “stability threat” in reflex modulation (Llewellyn et al., 1990; Haridas et al., 2005, 2006, 2008; Lamont and Zehr, 2006, 2007; Hoogkamer et al., 2012). Stability threat is defined as a context (e.g., a task) where posture can be more easily destabilized. For instance, compared to normal treadmill walking, responses evoked by stimulating the SP nerve were facilitated when walking with arms crossed (less stable) but were reduced when subjects were holding side bars (more stable) (Haridas et al., 2005, 2006, 2008). Walking on a beam also reduced the soleus H-reflex by ∼40% compared to treadmill walking, potentially to reduce destabilizing sensory feedback during precision walking (Llewellyn et al., 1990). However, these studies did not specifically assess left-right symmetry between the legs, despite its close association with walking stability. Notably, studies have reported greater left-right asymmetries during slow walking speeds, which are considered less stable, in both human subjects (Huijben et al., 2018) and spinal cats (Dambreville et al., 2015). More recently, it was shown that stepping at slow and fast speeds in spinal cats (cats with a spinal transection) increases left-right spatial asymmetry, and hindlimb cutaneous reflexes at these speeds are reduced compared to intermediate speeds (Hurteau et al., 2017).

To investigate the association between increased walking asymmetry, independent of speed, Hurteau et al. (2018) evoked hindlimb cutaneous reflexes during split-belt locomotion where the speeds of the left and right legs/limbs can be independently controlled, as shown in cats and humans (Forssberg et al., 1980; Dietz et al., 1994; Prokop et al., 1995; Reisman et al., 2005; Torres-Oviedo et al., 2011; Frigon et al., 2013, 2015, 2017; D’Angelo et al., 2014; Hoogkamer et al., 2014; Kuczynski et al., 2017; Hurteau and Frigon, 2018; Park et al., 2019). Studies in humans have demonstrated a return of left-right symmetry in some interleg variables, such as step length and double support periods, during prolonged split-belt locomotion, which has been termed an adaptation (Reisman et al., 2005; Finley et al., 2013, 2015). Cats, on the other hand, do not show this adaptation, exhibiting persistent left-right asymmetry during prolonged split-belt locomotion (Kuczynski et al., 2017), like human infants (Yang et al., 2005; Vasudevan et al., 2011). Thus, split-belt locomotion in cats allows for the study of reflexes in a condition of prolonged left-right asymmetry. Split-belt locomotion in cats produces a lateral shift of the center of mass toward the slower belt, potentially influencing stability (Park et al., 2019). It has been proposed that split-belt locomotion produces a limping gait, which can be considered unstable (Duysens et al., 2004). Hurteau et al. (2018) proposed that spinal networks perceive left-right spatial asymmetry as a threat to stability, resulting in a reduction in the gain of cutaneous inputs to prevent disruption of the locomotor pattern.

Currently, studies have mainly focused on the modulation of reflexes in the legs/hindlimbs evoked by stimulating nerves of the foot. However, stimulating cutaneous nerves in cats and humans evokes phase-dependent reflex responses in all four limbs, producing a whole-body response to a perturbation (Haridas and Zehr, 2003; Hurteau et al., 2018). Although the phase-dependence of these interlimb reflexes have been demonstrated during locomotion, less is known about their task- and speed-dependence. In the present study, we determined if and how split-belt locomotion modulates cutaneous reflex responses evoked by stimulating the SR and SP nerves in muscles of the four limbs. We used split-belt locomotion to induce a left-right asymmetry as a potential threat to stability. We hypothesized that split-belt locomotion reduces cutaneous reflexes to all four limbs in left-right asymmetric conditions.

## 2. Materials and methods

### 2.1. Animals and ethical approval

All procedures were approved by the Animal Care Committee of the Université de Sherbrooke (Protocol 442-18) in accordance with policies and directives of the Canadian Council on Animal Care. In the present study, we used eight cats (four males: HO, JA, KI, and TO; four females: AR, GR, KA, and PO) weighing 3.4–5.6 kg and 1–1.5 years of age at the time of experimentation. We followed the ARRIVE guidelines 2.0 for animal studies (Percie du Sert et al., 2020). In order to maximize the scientific output of each animal, they were used in other studies to investigate different scientific questions, some of which have been published (Lecomte et al., 2022, 2023; Merlet et al., 2022; Audet et al., 2023).

### 2.2. General surgical procedures

The implantation surgery was performed under aseptic conditions with sterilized equipment in an operating room. Before surgery, cats were sedated with an intramuscular (i.m.) injection of butorphanol (0.4 mg/kg), acepromazine (0.1 mg/kg), and glycopyrrolate (0.01 mg/kg). We injected a mixture of diazepam/ketamine (0.05 mg/kg, i.m.) for induction. Cats were anesthetized with isoflurane (1.5–3%) delivered with a mask and then with a flexible endotracheal tube. Anesthesia was maintained during surgery by adjusting isoflurane concentration as needed and by monitoring cardiac and respiratory rates. Body temperature was maintained constant (37 ± 0.5°C) using a water-filled heating pad placed under the animal and an infrared lamp placed ∼50 cm over it. At the end of surgery, we injected subcutaneously (s.c.) an antibiotic (cefovecin, 0.1 ml/kg) and a fast-acting analgesic (buprenorphine, 0.01 mg/kg). We also taped a fentanyl (25 μg/h) patch to the back of the animal 2–3 cm rostral to the base of the tail for prolonged analgesia, which we removed 4–5 days later. After surgery, cats were placed in an incubator and closely monitored until they regained consciousness. We administered another dose of buprenorphine ∼7 h after surgery. When cats concluded other studies, they were administered a lethal dose of pentobarbital (120 mg/kg) through the left or right cephalic vein.

### 2.3. Electromyography and nerve stimulation

To record the electrical activity of muscles, we directed pairs of Teflon-insulated multistrain fine wires (AS633; Cooner Wire) subcutaneously from two head-mounted 34-pin connectors (Omnetics Connector). Two wires, stripped of 1–2 mm of insulation, were sewn into the belly of selected forelimb/hindlimb muscles for bipolar recordings. The head-mounted connectors were fixed to the skull using dental acrylic and four to six screws. We verified electrode placement during surgery by stimulating each muscle through the appropriate head connector channel to assess the biomechanically desired muscle contraction. During experiments, electromyography (EMG) signals were pre-amplified (×10, custom-made system), bandpass filtered (30–1,000 Hz) and amplified (100–5,000×) using a 16-channel amplifier (model 3500; AM Systems). EMG data were digitized (5,000 Hz) with a National Instruments card (NI 6032E), acquired with custom-made acquisition software and stored on computer. Five forelimb muscles were implanted bilaterally: biceps brachii (BB, elbow and shoulder flexor), the long head of the triceps brachii (TRI, elbow extensor), latissimus dorsi (LD, shoulder retractor), extensor carpi ulnaris (ECU, wrist dorsiflexor) and flexor carpi ulnaris (FCU, wrist plantarflexor). Ten hindlimb muscles were implanted bilaterally: anterior sartorius (SRT, hip flexor and knee extensor), semitendinosus (ST, knee flexor and hip extensor), vastus lateralis (VL, knee extensor), iliopsoas (IP, hip flexor), biceps femoris posterior (BFP, hip extensor and knee flexor), biceps femoris anterior (BFA, hip extensor), lateral gastrocnemius (LG, ankle extensor and knee flexor), soleus (SOL, ankle extensor), medial gastrocnemius (MG, ankle extensor and knee flexor), and tibialis anterior (TA, ankle flexor).

**FIGURE 1:**
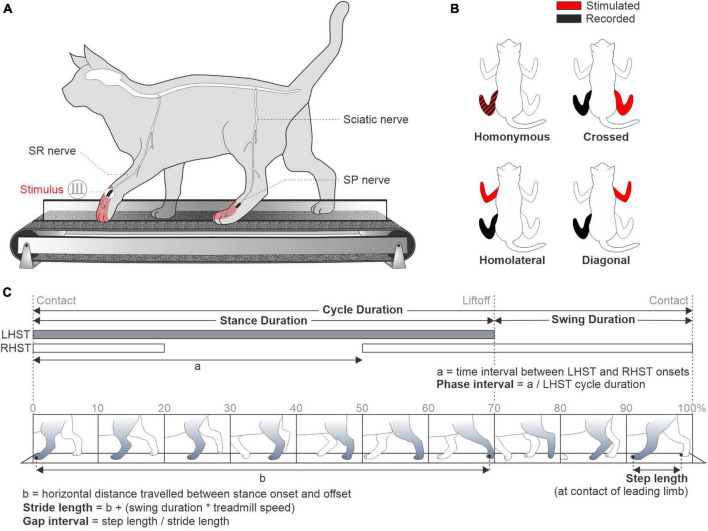
Experimental set-up and spatiotemporal parameters. **(A)** Experimental design illustrating a cat walking on a split-belt treadmill. The superficial radial (SR) and peroneal (SP) nerves were electrically stimulated. **(B)** The stimulated and recorded limbs are displayed in red and black, respectively. **(C)** Spatial and temporal parameters of the left fore- and hindlimb during a locomotor cycle divided in 10 phases (0–100%). LHST, left hindlimb stance; RHST, right hindlimb stance.

For bipolar nerve stimulation, pairs of Teflon-insulated multistrain fine wires (AS633; Cooner Wire) were passed through a silicon tubing. A horizontal slit was made in the tubing and wires within the tubing were stripped of their insulation. The ends protruding through the cuff were knotted to hold the wires in place and glued. The ends of the wires away from the cuff were inserted into four-pin connectors (Hirose or Samtec) and fixed to the skull using dental acrylic. Cuff electrodes were directed subcutaneously from head-mounted connectors to the left and right SP and SR nerves at the ankle and wrist, respectively, which are purely cutaneous at these levels (Bernard et al., 2007; Figure 1A).

### 2.4. Experimental design

We trained cats to step on a treadmill with two independently controlled belts 120 cm long and 30 cm wide (Bertec) for 2–3 weeks in a progressive manner, first for a few steps and then for several consecutive minutes, using food and affection as rewards. Once cats could perform 3–4 consecutive minutes, we started the experiments. In the tied-belt conditions (equal left-right speeds), the Tied Slow and Tied Fast conditions refer to speeds of 0.4 ± 0.1 and 0.8 ± 0.1 m/s, respectively. The slow and fast speeds varied depending on the size of the cat and their ability to maintain these speeds for several consecutive minutes. Cat KA stepped at 0.3 and 0.7 m/s while cat KI stepped at 0.5 and 0.9 m/s. The other cats stepped at 0.4 and 0.8 m/s. The objective was to set a speed difference between those two conditions of 0.4 m/s. We applied the same speeds in split-belt conditions (unequal left-right speeds) with the slow and fast limbs stepping at 0.4 ± 0.1 and 0.8 ± 0.1 m/s, respectively. Both the left and right sides were used as the slow and fast sides.

During experiments, we delivered trains of electrical stimuli consisting of three 0.2 ms pulses at 300 Hz using a Grass S88 stimulator. At the start of the experiment, we determined the motor threshold, defined as the minimal intensity that elicited a small motor response in an ipsilateral flexor muscle (e.g., ST or TA) during the swing phase. We then set stimulation intensity at 1.2 times the motor threshold. Each locomotor condition lasted 5–8 min and consisted of ∼120 stimuli delivered pseudo-randomly every 2–4 locomotor cycles at varying delays, based on the onset of an extensor burst. For a given nerve stimulation, all data were collected in the four locomotor conditions within a single session. We characterized responses in muscles of the stimulated limb (homonymous), the opposite limb of the same girdle (crossed), the limb on the same side (homolateral) and the diagonal limb (diagonal) (Figure 1B).

### 2.5. Kinematic acquisition and analysis

During experiments, two cameras (Basler AcA640-100gm) were used to capture videos from the left and right sides of the animals (60 frames per second; 640 × 480 pixels spatial resolution). A custom-made LabVIEW program acquired the images and synchronized the cameras with EMG data. We analyzed kinematic data from videos off-line with a deep learning approach (DeepLabCut™; Mathis et al., 2018), which we recently validated in our cat model (Lecomte et al., 2021). We determined contact and liftoff of the four limbs by visual inspection in all locomotor conditions separately. Paw contact was defined as the first frame where the paw made visible contact with the treadmill surface. Paw liftoff was defined as the frame with the most caudal displacement of the toe. We measured cycle duration as the interval of time from successive paw contacts of the same limb (Figure 1C). Stance duration corresponded to the interval of time from contact to liftoff of the same limb, while swing duration was measured as cycle duration minus stance duration. We measured step length as the distance between the leading and trailing limb at stance onset of the leading limb (Hoogkamer et al., 2014). We measured stride length as the horizontal distance between contact and liftoff of the limb, added to the horizontal distance traveled by the treadmill during swing duration (Courtine et al., 2005; Thibaudier and Frigon, 2014). For these analyses, we only used cycles without a stimulation.

We quantified temporal interlimb coordination by measuring phase intervals between pairs of limbs, defined as the absolute interval of time between contacts of two limbs divided by the cycle duration of the reference limb (English, 1979; English and Lennard, 1982; Frigon et al., 2014; Thibaudier and Frigon, 2014; Thibaudier et al., 2017). We calculated phase intervals for four different pairs of limbs: homologous forelimbs, homologous hindlimbs, left homolateral limbs, and diagonal coupling between the left forelimb and right hindlimb. The reference limb was always the left forelimb, except for homologous hindlimb coupling where it was the left hindlimb. Phase intervals with values of 0° and 360° indicate a strict in-phase coordination whereas a value of 180° indicates a strict out-of-phase alternation between two limbs. We quantified spatial interlimb coordination by measuring gap intervals between pairs of limbs, defined as the step length divided by stride length of the reference limb (Abourachid et al., 2007; Thibaudier et al., 2017). Gap intervals were only calculated for homologous forelimb and hindlimb couplings. Gap intervals with values of 0° and 360° indicate, on the horizontal axis, that the two limbs contact the treadmill surface at the same location. A value of 180° indicates that the two limbs contact the surface of the treadmill at a distance corresponding to half of the stride length. Phase and gap intervals were then multiplied by 360 and expressed in degrees to illustrate their continuous nature and possible distributions (English and Lennard, 1982).

To assess if the locomotor pattern deviates from perfect left-right symmetry on a step-by-step basis for homologous forelimb and hindlimb couplings (Hurteau et al., 2017), we calculated temporal and spatial symmetry indexes by measuring the relative deviation of the phase and gap interval from a perfect symmetry of 180°, respectively. Symmetry indexes were then multiplied by 100 and expressed as a percentage.

### 2.6. Reflex analysis

**FIGURE 2:**
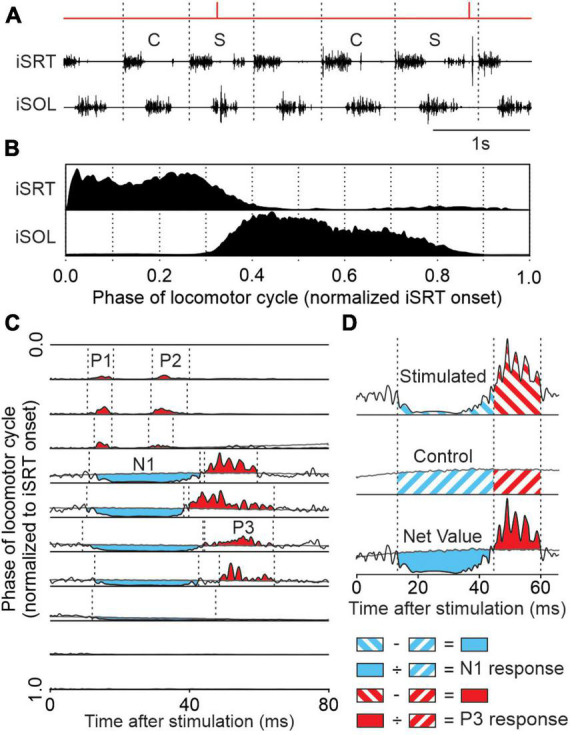
Reflex analysis. **(A)** We tagged cycles as stimulated (S) when a stimulus fell within the cycle or control (C) if it was not preceded by a stimulated cycle. **(B)** We averaged control cycles for the different muscles normalized to the ipsilateral sartorius (iSRT) onset. Each normalized cycle was separated into 10 bins to provide a baseline locomotor EMG (blEMG) in each phase. **(C)** Stimulated cycles (black traces) were averaged into 1 of 10 bins and superimposed on the blEMG (gray traces). This allowed us to determine positive (in red) and negative (in blue) responses. **(D)** Onsets and offsets of responses, defined as a prominent positive or negative deflection away from the blEMG, were determined visually. In each phase, the blEMG occurring in the same time window as the response was subtracted from the response in the stimulated cycles to provide a net reflex value. This value is then divided by the blEMG occurring in the same time window giving N1 and P3 amplitudes for this muscle (ipsilateral soleus).

We describe the reflex analysis in several of our publications (Hurteau et al., 2017, 2018; Hurteau and Frigon, 2018; Merlet et al., 2020, 2021). We first define locomotor cycles from successive onsets of the left sartorius and group them as stimulated (i.e., cycles with stimulation) or control (i.e., cycles without stimulation) (Figure 2A). Sections where the cat stepped irregularly were removed from analysis based on EMG and video data. The locomotor cycle was then divided into 10 subphases of equal duration. Control cycles were averaged and rectified to provide a baseline locomotor EMG (blEMG) in each phase (Figure 2B), providing an indication of the excitability level of the motor pool during stimulation. Stimulated cycles were averaged, rectified and separated into 10 phases according to the time when stimulation occurred within the cycle. The blEMG was then superimposed with reflex responses (i.e., stimulated cycles) for the 10 locomotor phases (Figure 2C). To characterize response onsets and offsets, defined as prominent positive or negative deflections away from the blEMG, we set windows using previous studies as guidelines (Duysens and Stein, 1978; Duysens and Loeb, 1980; Pratt et al., 1991; Loeb, 1993; Hurteau et al., 2016, 2017, 2018) with 97.5% confidence intervals. We termed short-latency (7–18 ms) excitatory and inhibitory responses as P1 and N1 responses, respectively, based on the terminology introduced by Duysens and Loeb (1980). Responses in the crossed, homolateral, and diagonal limbs that had an onset ≤18 ms were classified as P1 or N1, as the minimal latency for spino-bulbo-spinal reflexes in the cat is 18 ms (Shimamura and Livingston, 1963). Mid-latency (19–34 ms) excitatory and inhibitory responses were termed P2 and N2, respectively. Longer-latency (35–60 ms) excitatory and inhibitory responses were termed P3 and N3, respectively.

The EMG of reflex responses was integrated and then subtracted from the integrated blEMG in the same time window to provide a net reflex value (Figure 2D). This net reflex value was then divided by the integrated blEMG value to evaluate reflex responses. This division identifies if changes in reflex responses across the cycle or according to locomotor condition are independent of changes in blEMG activity (Matthews, 1986; Frigon and Rossignol, 2007, 2008a,b; Frigon et al., 2009; Hurteau et al., 2017, 2018; Hurteau and Frigon, 2018). The modulation of reflexes by phase and task (tied-belt and split-belt locomotion) was illustrated by normalizing reflex responses in each muscle independently to the maximal value (expressed as a percentage) obtained in one of the four locomotor conditions. To better evaluate the effect of the locomotor condition on the phase-dependent modulation, we calculated a reflex modulation index by measuring the difference between the largest and smallest responses out of the 10 phases for each locomotor condition (Hurteau et al., 2017, 2018; Hurteau and Frigon, 2018). Reflex indexes were then normalized in each muscle to the maximal value obtained in one of the four conditions. In some muscles, we obtained a reflex index superior to 100% because of inhibitory responses in some phases and excitatory responses in others. This index allows us to measure and compare the depth of reflex modulation across conditions.

### 2.7. Statistical analysis

We performed statistical tests with IBM SPSS Statistics V26 (IBM Corp., Armonk, NY, USA). We quantified reflex responses for five forelimb muscles (BB, ECU, FCU, LD, and TRI) and ten hindlimb muscles (BFA, BFP, IP, LG, MG, SRT, SOL, ST, TA, and VL). As both left and right SR nerves evoked responses, we treated them separately and pooled them for statistical analysis, as done previously (Hurteau et al., 2018). We applied the same approach for left and right SP nerves. This gave us a total of 16 SR and 16 SP nerve stimulations in 8 cats, respectively. However, only evoked responses are used and pooled for statistics according to each muscle. To evaluate whether homonymous, crossed, homolateral, and diagonal responses were modulated by phase in the four locomotor conditions, a one-factor (phase) repeated-measures ANOVA was performed on all responses (P1, P2, P3, N1, N2, and N3) for a given muscle. To compare reflex modulation indexes between locomotor conditions, we performed a one-factor (condition) repeated-measures ANOVA followed by pairwise comparisons (Student’s test) if a significant main effect was found. Even with a small sample size, assumptions of normality were considered respected as we expected that our sample outcomes came from a normal population distribution.

For statistical analysis on interlimb coordination, all eight cats were used. For temporal (cycle, stance, and swing durations) and spatial (step and stride lengths) parameters, pairwise comparisons were used if there was a main effect of the one-factor (condition) repeated-measures ANOVA. For phase and gap intervals, we performed a circular analysis with a MATLAB toolbox for circular statistics (Berens, 2009). We calculated the resultant vector length (*r*) to quantify the circular spread of values around the mean. A value close to 1 means that the data sample is concentrated around the mean direction, whereas a value of 0 indicates a uniform distribution. Rayleigh’s test was then performed to detect unimodal deviation from uniformity from the resultant vector length. To determine whether mean directions between two locomotor conditions were different, we performed Watson-Williams’ test for the mean phase and gap intervals (Watson and Williams, 1956; Stephens, 1969). We performed a one-factor (condition) repeated-measures ANOVA on temporal and spatial symmetry indexes of the phase/gap intervals for the forelimb and hindlimb couplings. If there was a significant effect of condition detected by the one-factor repeated-measures ANOVA, we performed pairwise comparisons without adjustments. As discussed in one of our previous studies (Hurteau and Frigon, 2018), we did not correct for multiple comparisons following Rothman’s recommendation (Rothman, 1990) to avoid type II errors. Statistical significance for all tests was set at *P* < 0.05.

## 3. Results

### 3.1. Characteristics of the locomotor pattern during tied-belt and split-belt locomotion

**FIGURE 3:**
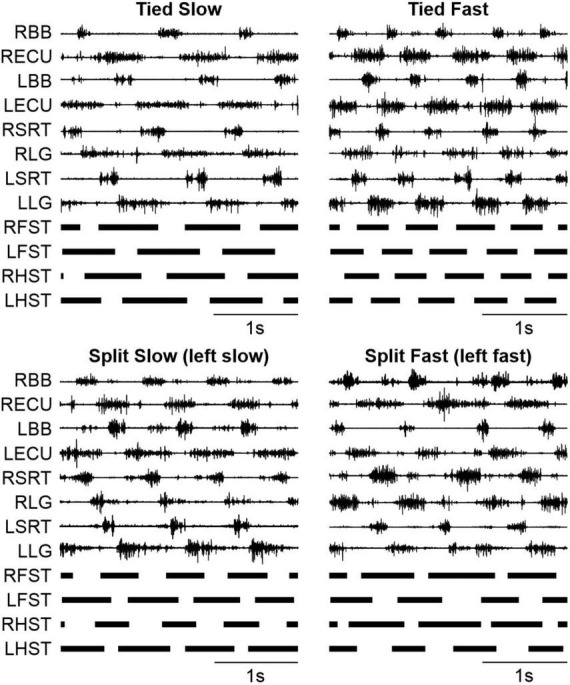
Modulation of the locomotor pattern during tied-belt and split-belt locomotion in a single cat. The figure shows EMG activities for selected forelimb (BB and ECU) and hindlimb (SRT and LG) muscles bilaterally along with the stance phases (thick black bars) for all four limbs. The vertical and horizontal scales are the same for a given muscle in all four panels. L, left; R, right; LG, lateral gastrocnemius; SRT, anterior sartorius; ECU, extensor carpi ulnaris; BB, biceps brachii; RFST, right forelimb stance; LFST, left forelimb stance; RHST, right hindlimb stance; LHST, left hindlimb stance.

To study cutaneous reflexes and their modulation in different locomotor conditions, we first investigated changes in the locomotor pattern. Figure 3 shows EMG of selected forelimb (BB and ECU) and hindlimb (SRT and LG) muscles bilaterally along with stance phases of the four limbs in a representative cat at two speeds during tied-belt locomotion (Tied Slow and Tied Fast) and two left-right speeds in split-belt locomotion (Split Slow and Split Fast). Split Slow and Split Fast refer to the ipsilateral limb when it was stepping on the slow and fast belts, respectively. In the example, the left side is slow and fast in Split Slow and Split Fast conditions, respectively. In tied-belt conditions, increasing treadmill speed reduced stance phases and EMG extensor burst durations bilaterally. Larger burst amplitudes were also apparent in Tied Fast compared to Tied Slow in most muscles. During split-belt locomotion, ipsilateral stance/extensor burst durations were longer on the slow side while swing/flexor burst durations were longer on the fast side. We also observed greater and smaller EMG burst amplitudes for limb muscles stepping on the slow and fast sides, respectively.

**FIGURE 4:**
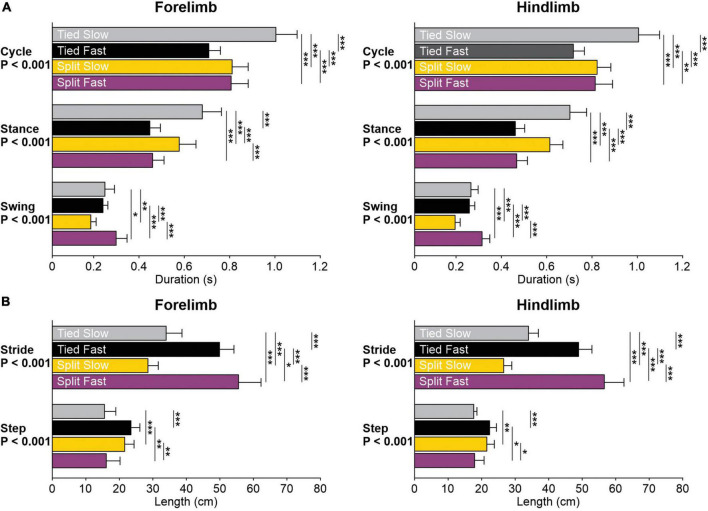
Temporal and spatial parameters during tied-belt and split-belt locomotion across animals. **(A)** Cycle, stance, and swing durations are shown for the left forelimb and hindlimb. **(B)** Step and stride lengths are shown for the left forelimb and hindlimb. Each bar is the mean ± SD from 8 cats. Asterisks indicate significant differences between locomotor conditions (pairwise comparisons): *P < 0.05; **P < 0.01; ***P < 0.001.

To determine how the locomotor conditions affect the temporal structure of the step cycle, we measured cycle, stance and swing durations in the left forelimb and left hindlimb across animals (Figure 4A). Locomotor condition significantly affected cycle (Forelimb, *P* = 1.4 × 10^–9^; Hindlimb, *P* = 5.0 × 10^–10^), stance (Forelimb, *P* = 1.8 × 10^–12^; Hindlimb, *P* = 1.8 × 10^–14^) and swing (Forelimb, *P* = 1.4 × 10^–7^; Hindlimb, *P* = 2.3 × 10^–9^) durations. We observed similar adjustments in the fore- and hindlimbs for cycle duration with Tied Slow > Split Slow and Split Fast > Tied Fast; stance duration with Tied Slow > Split Slow > Tied Fast and Split Fast; and swing duration with Split Fast > Tied Slow and Tied Fast > Split Slow. These adjustments confirm those observed in the fore- and/or hindlimbs during quadrupedal or hindlimb-only locomotion in intact and spinal cats (D’Angelo et al., 2014; Frigon et al., 2015; Hurteau and Frigon, 2018).

To determine how the locomotor conditions affect the spatial structure of the step cycle, we measured stride and step lengths in the left forelimb and left hindlimb across animals (Figure 4B). Locomotor condition significantly affected stride (Forelimb, *P* = 3.8 × 10^–13^; Hindlimb, *P* = 1.2 × 10^–15^) and step (Forelimb, *P* = 7.0 × 10^–7^; Hindlimb, *P* = 1.9 × 10^–4^) lengths. We observed similar adjustments in the fore- and hindlimbs for stride length with Split Fast > Tied Fast > Tied Slow > Split Slow. For step length, the forelimbs and hindlimbs also adjusted similarly with Tied Fast and Split Slow > Tied Slow and Split Fast.

### 3.2. Interlimb coordination during tied-belt and split-belt locomotion

**FIGURE 5:**
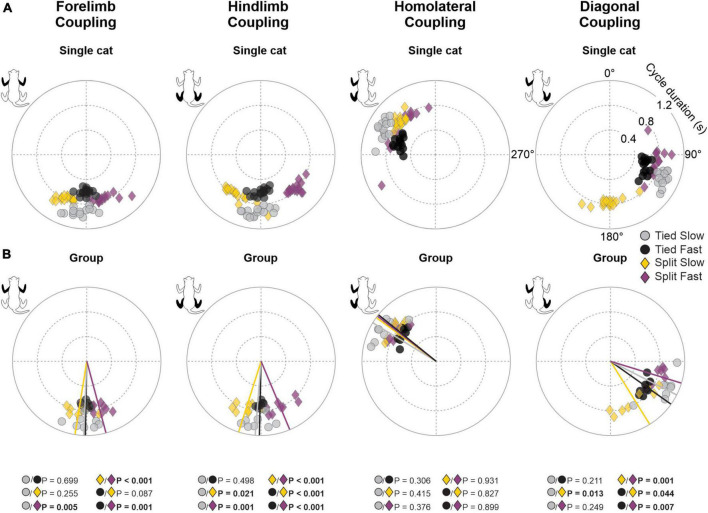
Phase intervals between limb pairs during tied-belt and split-belt locomotion in a single cat and across animals. Circular plots show phase intervals expressed in degrees around the circumference while cycle durations are plotted in radii. **(A)** Each data point represents a locomotor cycle in a single cat. **(B)** Each data point represents the average of 7–21 locomotor cycles from the eight cats. Straight lines starting from the center of the circle to the circumference display mean directions for each locomotor condition. *P*-values in bold indicate whether mean directions between two locomotor conditions were significantly different for group data (Watson-Williams’ test).

To assess temporal interlimb coordination, we measured phase intervals between four limb pairs. As we can see for a representative cat, phase interval values were clustered for a given condition, indicating consistent step-by-step phasing between limbs (Figure 5A). Rayleigh’s test performed for each cat confirmed that phase intervals of all limb couplings were not randomly distributed for all locomotor conditions, with *r* values ranging from 0.89 to 1.0 (Table 1). However, we did observe significant shifts in phase intervals between conditions for three limb pairs (Forelimb coupling, *P* = 3.4 × 10^–4^; Hindlimb coupling, *P* = 6.0 × 10^–7^; Diagonal coupling, *P* = 7.3 × 10^–4^) across animals (Figure 5B). As stated in section “2. Materials and methods,” the reference limb was always the left forelimb, except for homologous hindlimb coupling where it was the left hindlimb. Functionally, when a condition displayed greater values compared to another, contact of the non-reference limb occurred later relative to the reference limb.

**TABLE 1:** Circular statistics on phase intervals during tied- and split-belt locomotion.

Cat identification		AR	GR	HO	JA	KA	KI	PO	TO
Forelimb coupling	Tied slow	**0.97**	**0.97**	**0.98**	**0.93**	**0.99**	**0.96**	**0.98**	**0.96**
	Tied fast	**1.00**	**1.00**	**0.99**	**0.97**	**0.99**	**1.00**	**1.00**	**0.98**
	Split slow	**0.97**	**0.99**	**0.99**	**0.98**	**0.99**	**0.99**	**0.98**	**0.94**
	Split fast	**0.92**	**0.99**	**0.96**	**0.96**	**0.98**	**1.00**	**0.98**	**0.98**
Hindlimb coupling	Tied slow	**0.97**	**0.98**	**0.98**	**0.89**	**0.98**	**0.96**	**0.99**	**0.96**
	Tied fast	**0.99**	**0.99**	**1.00**	**0.99**	**0.99**	**1.00**	**1.00**	**0.99**
	Split slow	**0.99**	**0.97**	**0.96**	**0.96**	**0.97**	**0.98**	**0.98**	**0.94**
	Split fast	**0.98**	**0.99**	**0.97**	**0.99**	**0.99**	**0.99**	**0.99**	**0.99**
Homolateral coupling	Tied slow	**0.98**	**0.98**	**0.96**	**0.98**	**0.99**	**0.99**	**0.97**	**0.97**
	Tied fast	**1.00**	**0.99**	**0.99**	**0.94**	**0.99**	**0.99**	**1.00**	**0.97**
	Split slow	**0.97**	**0.98**	**0.97**	**0.97**	**0.99**	**0.99**	**0.98**	**0.97**
	Split fast	**0.94**	**0.94**	**0.97**	**0.96**	**0.91**	**0.97**	**0.98**	**0.92**
Diagonal coupling	Tied slow	**0.98**	**0.99**	**0.99**	**0.94**	**0.99**	**0.97**	**0.96**	**0.97**
	Tied fast	**0.99**	**0.99**	**0.99**	**0.94**	**0.98**	**0.99**	**1.00**	**0.97**
	Split slow	**0.91**	**0.96**	**0.96**	**0.93**	**0.97**	**0.97**	**0.95**	**0.97**
	Split fast	**0.96**	**0.95**	**0.97**	**0.95**	**0.96**	**0.99**	**0.98**	**0.97**

The table shows *r* values (resultant vector length) of phase intervals for the four limb couplings in each cat (*n* = 8) for all locomotor conditions. A value close to 1 means that the data sample is concentrated around the mean direction, whereas a value of 0 indicates a uniform distribution. Bold *r* values indicate a common mean direction of phase intervals between two limbs (significant Rayleigh’s test). For each condition, we averaged 7–21 cycles per cat.

**FIGURE 6:**
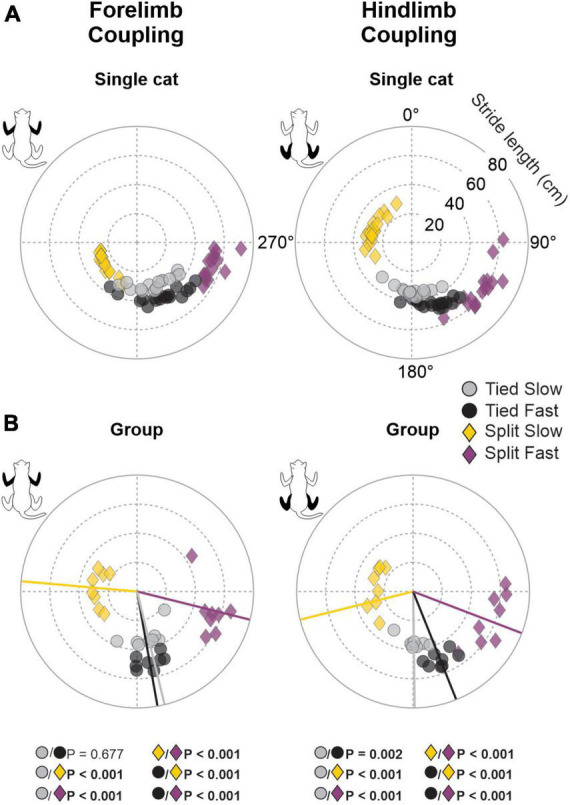
Gap intervals for forelimb and hindlimb couplings during tied-belt and split-belt locomotion in a single cat and across animals. Circular plots show gap intervals expressed in degrees around the circumference stride lengths are plotted in radii. **(A)** Each data point represents a locomotor cycle in a single cat. **(B)** Each data point represents the average of 7–21 locomotor cycles from the eight cats. Straight lines starting from the center of the circle to the circumference display mean directions for each locomotor condition. *P*-values in bold indicate whether mean directions between two locomotor conditions were significantly different for group data (Watson-Williams’ test).

To quantify spatial coordination between homologous limbs at the shoulder and pelvic girdles, we measured gap intervals. Compared to temporal phasing, spatial phasing between limbs was more variable on a step-by-step basis, as shown for a single cat (Figure 6A). This depended on the cat and the limb coupling (Table 2). For instance, hindlimb coupling was relatively strong across cats and conditions with a mean r value of 0.89 ± 0.09 and a range of 0.62–0.99. Forelimb coupling was also relatively strong with a mean *r* value of 0.86 ± 0.14 but with a wider range of 0.47–0.99. In the Tied Slow condition, some cats step more toward the front of the treadmill resulting in greater variability. Across animals, we observed significant shifts in gap intervals between conditions for forelimb (*P* = 1.5 × 10^–13^) and hindlimb (*P* = 1.3 × 10^–13^) couplings (Figure 6B). Similar observations were found between those two homologous couplings. In Tied Slow and Tied Fast, values were around 180°, while in Split Slow and Split Fast, they were mirror images at 270° and 90°, respectively. This means that unlike tied-belt locomotion, the non-reference limb contacted the treadmill at a distance corresponding to 1/3 and 2/3 of the reference limb’s stride length. Except for Tied Slow versus Tied Fast in forelimb coupling, we observed significant differences between all conditions for the two homologous couplings.

**TABLE 2:** Circular statistics on gap intervals during tied- and split-belt locomotion.

Cats identification		AR	GR	HO	JA	KA	KI	PO	TO
Forelimb coupling	Tied slow	**0.80**	**0.86**	**0.91**	**0.61**	**0.92**	**0.71**	**0.69**	0.47
	Tied fast	**0.97**	**0.98**	**0.98**	**0.86**	**0.92**	**0.97**	**0.99**	**0.95**
	Split slow	**0.96**	**0.84**	**0.91**	0.62	**0.95**	**0.95**	**0.62**	**0.70**
	Split fast	**0.91**	**0.96**	**0.95**	**0.81**	**0.97**	**0.98**	**0.97**	**0.88**
Hindlimb coupling	Tied slow	**0.83**	**0.93**	**0.97**	**0.62**	**0.95**	**0.83**	**0.88**	**0.76**
	Tied fast	**0.97**	**0.98**	**0.99**	**0.89**	**0.97**	**0.98**	**0.99**	**0.95**
	Split slow	**0.74**	**0.87**	**0.87**	**0.72**	**0.93**	**0.89**	**0.91**	**0.76**
	Split fast	**0.93**	**0.97**	**0.90**	**0.88**	**0.96**	**0.96**	**0.94**	**0.90**

The table shows *r* values (resultant vector length) of gap intervals for forelimb and hindlimb couplings in each cat (*n* = 8) for all locomotor conditions. A value close to 1 means a data sample concentrated around the mean direction, whereas a value of 0 indicates uniform distribution. Bold *r* values indicate a common mean direction of gap intervals for a limb coupling (significant Rayleigh’s test). For each condition, we averaged 7–21 cycles per cat.

**FIGURE 7:**
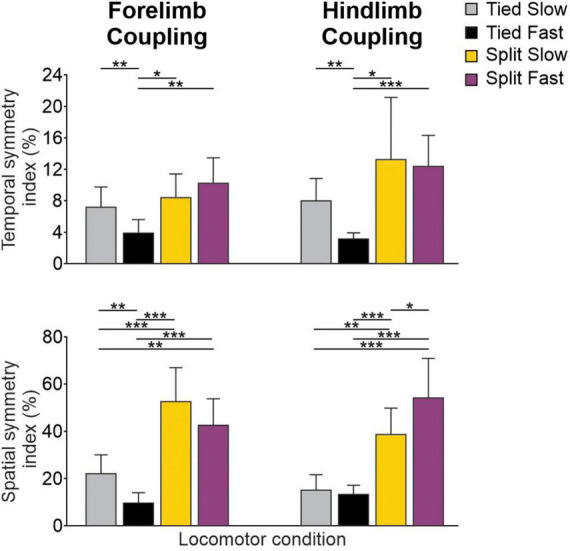
Spatiotemporal coordination for forelimb and hindlimb couplings during tied-belt and split-belt locomotion across animals. Histograms show temporal and spatial symmetry indexes in the four locomotor conditions across animals. Smaller percentage values indicate greater left-right symmetry between forelimb/hindlimb pairs. Each vertical bar is the mean ± SD from eight cats. Asterisks indicate significant differences between conditions (pairwise comparisons): *P < 0.05; **P < 0.01; ***P < 0.001.

To evaluate the consistency of bilateral coordination at the shoulder and pelvic girdles across animals, we measured temporal and spatial symmetry indexes for homologous couplings (Figure 7; Thibaudier and Frigon, 2014; Hurteau and Frigon, 2018). Smaller symmetry index percentages reflect greater left-right symmetry between limbs on a step-by-step basis. As we can see, the spatial consistency of left-right symmetry is more affected than the temporal one. The temporal symmetry index only differed significantly for Tied Fast compared to all other conditions for forelimb and hindlimb couplings, with a mean percentage about half of the others, indicating greater consistency. The spatial consistency of left-right symmetry was considerably greater (smaller percentages) during tied-belt locomotion compared to split-belt locomotion for both forelimb and hindlimb couplings. Thus, during split-belt locomotion, left-right spatial coordination is more asymmetric on a step-by-step basis in the fore- and hindlimbs compared to tied-belt locomotion, as shown previously for the hindlimbs of intact and spinal cats (D’Angelo et al., 2014; Frigon et al., 2015; Hurteau and Frigon, 2018).

### 3.3. Cutaneous reflex responses in forelimb muscles

**FIGURE 8:**
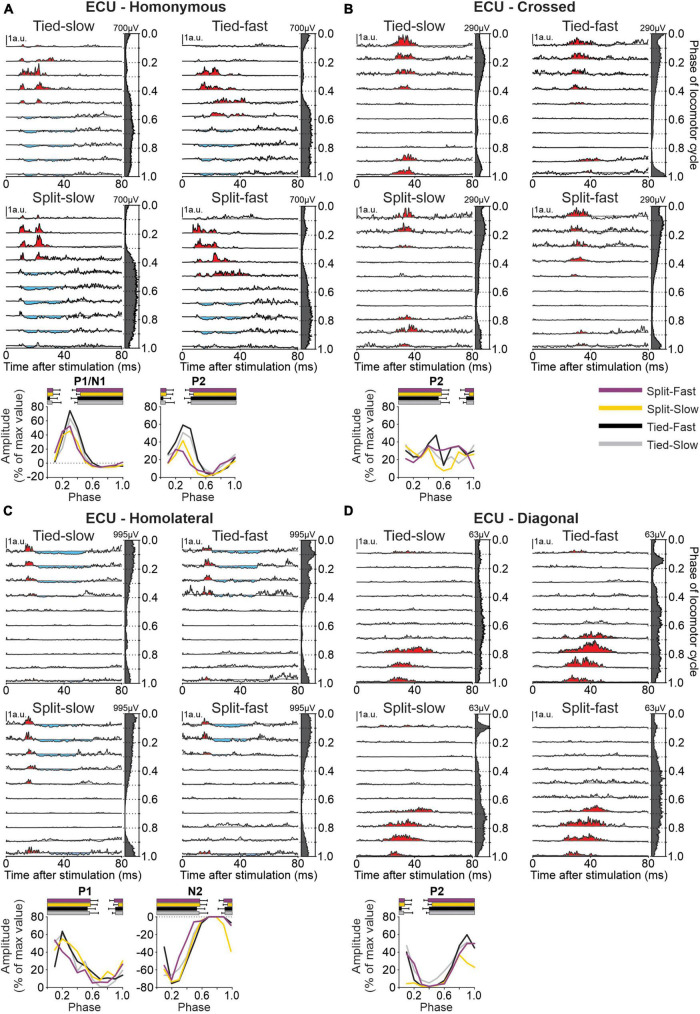
Intra- and interlimb reflexes in a forelimb muscle during tied-belt and split-belt locomotion. Each panel shows reflex responses in 10 phases of the cycle evoked in the extensor carpi ulnaris (ECU) in a single cat by **(A)** stimulating the homonymous superficial radial (SR) nerve, **(B)** the crossed SR, **(C)** the homolateral superficial peroneal (SP) nerve, and **(D)** the diagonal SP in the four locomotor conditions. Black traces are averaged cycles that received a stimulation (*n* = 4–26 stimuli per phase) while gray traces are averaged cycles (blEMG) without stimulation (*n* = 88–140 cycles). Scale bars are shown in arbitrary units (a.u.) and are the same across phases and conditions for each limb. Aligned vertically on the right side of each panel is the averaged rectified EMG of the ECU in the normalized cycle. At the bottom of each panel, the amplitude of short- and longer-latency responses is shown for the group within a normalized cycle to illustrate phase-dependent modulation. SR stimulation evoked homonymous (*n* = 12 P1/N1 in 8/8 cats and *n* = 9 P2 in 6/8 cats) and crossed (*n* = 8 P2 in 5/8 cats) responses while SP nerve stimulation evoked homolateral (*n* = 9 P1 in 7/8 cats and *n* = 12 N2 in 8/8 cats) and diagonal (*n* = 7 P2 in 5/8 cats) responses. Horizontal bars at the top represent the period of ECU activity for each locomotor condition (*n* = 7–19 control cycles) for pooled data.

To determine phase-, speed- and task-dependent modulation of forelimb cutaneous reflexes during quadrupedal locomotion, we recorded EMG from five forelimb muscles: BB, ECU, FCU, LD, and TRI. As an example of cutaneous reflexes in a forelimb muscle, Figure 8 shows responses in ECU evoked by stimulating the homonymous SR, crossed SR, homolateral SP and diagonal SP nerves in all four locomotor conditions. We selected the ECU, a wrist extensor and adductor, that is active during the stance phase (Drew and Rossignol, 1987; Krouchev et al., 2006; Hurteau et al., 2018) because reflex responses from stimulating all four limbs were consistently evoked in this muscle. Stimulating the homonymous SR nerve (Figure 8A) evoked N1 and P2 responses when ECU was active and P1 responses when inactive, consistent with previous studies (Drew and Rossignol, 1987; Hurteau et al., 2018). We found a significant phase-dependent modulation of P1/N1 responses for all four locomotor conditions in pooled data but not for P2. Crossed P2 responses (Figure 8B) observed during ECU activity were not significantly modulated in the different locomotor conditions. Homolateral responses evoked in ECU by SP nerve stimulation consisted of P1 responses followed by N2 responses (Figure 8C) while diagonal responses consisted only of P2 responses (Figure 8D). All homolateral and diagonal responses were significantly modulated with phase for pooled data in the four locomotor conditions.

**FIGURE 9:**
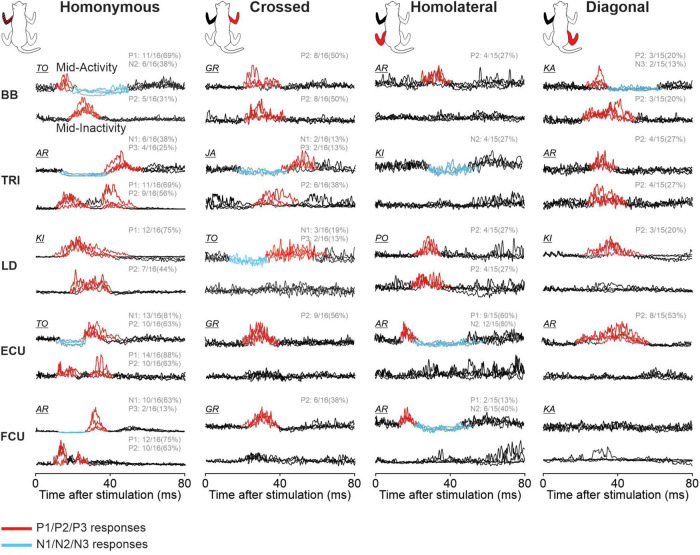
Phase-dependent modulation of forelimb cutaneous reflexes during tied-belt and split-belt locomotion. Homonymous, crossed, homolateral, and diagonal responses are shown for biceps brachii (BB), triceps brachii (TRI), latissimus dorsi (LD), extensor (ECU), and flexor (FCU) carpi ulnaris muscles. The limbs stimulated and recorded in the cat diagram are displayed in red and black, respectively. Each black trace represents averaged stimulated cycles (*n* = 6–32 cycles) for a locomotor condition during a muscle’s period of mid-activity or mid-inactivity. The four locomotor conditions are superimposed for comparisons in a representative cat and optimized for display. Evoked responses are highlighted in red for positive and blue for negative. The fraction for each response indicates the proportion of pooled data evoking the same pattern of response in all locomotor conditions.

Figure 9 illustrates reflex responses obtained for the five forelimb muscles with stimulation during mid-activity or mid-inactivity for a representative cat. Due to inter-animal variability (Loeb, 1993; Frigon, 2011), not all cats presented the same response pattern. Only responses reflecting the general observed pattern for the group determined by visual inspection based on 97.5% confidence intervals are shown (see section “2. Materials and methods”). Hence the cat used as an example for each response may differ. Responses were averaged for each locomotor condition separately and superimposed for all limb responses. Mid-activity and mid-inactivity periods were selected at ±7.5% around the midpoint of the activity and inactivity periods of a given muscle, respectively. A response was considered valid when the mean of the stimulated cycles deviated sufficiently from the mean of control cycles at confidence intervals of 97.5%. Positive and negative responses are highlighted in red and blue, respectively. Responses are optimized for display to highlight the pattern of responses. The fraction in each representation indicates the proportion of pooled data evoking the same pattern of response in all locomotor conditions. For example, 11/16 (69%) and 6/16 (38%) for homonymous BB means that of the 16 SR stimulations, 11 and 6 evoked P1 and N2 responses, respectively, in all four locomotor conditions during the muscle’s mid-activity. Note that the total number in each muscle may differ because some muscles were not implanted in all cats and some did not provide an adequate recording. Table 3 summarizes the phase-dependent modulation of responses in the five forelimb muscles for pooled data.

**TABLE 3:** Phase-dependent modulation of responses evoked by cutaneous inputs from the four limbs during tied-belt and split-belt locomotion in forelimb muscles.

		Homonymous				Crossed	Homolateral			Diagonal
		**P1/N1**	**P2**	**P3**	**N2**	**P2**	**P1**	**P2**	**N2**	**P2**
BB	Tied slow	5.7 × 10^–3^	8.4 × 10^–1^		2.0 × 10^–2^	1.9 × 10^–1^		3.1 × 10^–1^		
	Tied fast	6.5 × 10^–7^	4.2 × 10^–1^		4.9 × 10^–2^	5.0 × 10^–2^		2.6 × 10^–1^		
	Split slow	6.7 × 10^–3^	3.2 × 10^–1^		2.7 × 10^–2^	4.8 × 10^–2^		3.4 × 10^–1^		
	Split fast	4.0 × 10^–3^	6.6 × 10^–1^		8.7 × 10^–2^	5.7 × 10^–3^		3.4 × 10^–1^		
TRI	Tied slow	5.6 × 10^–7^	6.4 × 10^–4^	2.2 × 10^–1^		1.5 × 10^–1^			1.7 × 10^–2^	1.6 × 10^–1^
	Tied fast	1.2 × 10^–7^	1.9 × 10^–3^	3.5 × 10^–1^		1.6 × 10^–1^			4.8 × 10^–2^	3.0 × 10^–1^
	Split slow	1.4 × 10^–4^	4.9 × 10^–3^	5.0 × 10^–1^		2.2 × 10^–1^			1.8 × 10^–1^	3.6 × 10^–1^
	Split fast	5.6 × 10^–4^	1.0 × 10^–4^	5.2 × 10^–1^		2.7 × 10^–1^			1.3 × 10^–1^	3.2 × 10^–1^
LD	Tied slow	1.2 × 10^–5^	2.8 × 10^–2^					3.4 × 10^–2^		
	Tied fast	5.9 × 10^–7^	1.4 × 10^–2^					1.4 × 10^–1^		
	Split slow	7.9 × 10^–5^	4.4 × 10^–3^					2.1 × 10^–1^		
	Split fast	6.0 × 10^–5^	9.7 × 10^–2^					6.9 × 10^–2^		
ECU	Tied slow	1.4 × 10^–8^	1.1 × 10^–2^			5.0 × 10^–1^	2.2 × 10^–4^		4.0 × 10^–10^	1.3 × 10^–2^
	Tied fast	1.5 × 10^–7^	1.0 × 10^–2^			4.0 × 10^–1^	1.1 × 10^–2^		1.5 × 10^–7^	1.0 × 10^–2^
	Split slow	2.2 × 10^–6^	6.0 × 10^–2^			1.2 × 10^–1^	2.6 × 10^–2^		2.8 × 10^–9^	9.2 × 10^–3^
	Split fast	2.6 × 10^–8^	2.0 × 10^–1^			3.8 × 10^–1^	1.3 × 10^–3^		9.9 × 10^–8^	3.1 × 10^–2^
FCU	Tied slow	2.9 × 10^–10^	3.1 × 10^–1^			2.3 × 10^–1^			8.5 × 10^–2^	
	Tied fast	5.7 × 10^–6^	3.6 × 10^–1^			2.4 × 10^–1^			6.7 × 10^–3^	
	Split slow	4.5 × 10^–6^	5.9 × 10^–1^			5.0 × 10^–1^			4.3 × 10^–2^	
	Split fast	4.6 × 10^–8^	7.2 × 10^–1^			7.9 × 10^–1^			4.0 × 10^–2^	

The table shows *P*-values (repeated-measures ANOVA) of phase-dependent modulation for all forelimb reflex responses (P1, P2, P3, N1, N2, and N3) in the four locomotor conditions for pooled data. Figure 9 provides details on the number of pooled data used each response. *P*-values in red indicate a significant phase dependent-modulation. BB, biceps brachii; TRI, triceps brachii; LD, latissimus dorsi; ECU, extensor carpi ulnaris; FCU, flexor carpi ulnaris.

Homonymous P1 or N1 responses were present in all five muscles and were significantly modulated in all four locomotor conditions. Homonymous P2 responses were also present in all five muscles, albeit less frequently, but were significantly phase modulated in all four conditions for TRI only. Homonymous P2 responses in BB and FCU showed no phase modulation in all four conditions. Homonymous P2 responses in LD were phase modulated in both tied conditions and Split Slow while those in ECU were phase modulated only in both tied conditions. Only TRI had homonymous P3 responses and they were not phase modulated. Only BB had homonymous N2 responses and they were phase modulated in both tied-belt conditions and Split Slow. In the crossed forelimb, we observed P2 responses in four muscles, with the exception of LD, which were more frequent during the period of activity. We found significant phase modulation for BB in the two split-belt conditions only. Homolateral forelimb responses were more variable and almost exclusively found during the period of activity, with the exception of LD. ECU was the only muscle with homolateral P1 responses and these were phase modulated in all four conditions. BB and LD had homolateral P2 responses and these were phase modulated only for LD in Tied Slow. We observed homolateral N2 responses in TRI, ECU, and FCU. Homolateral N2 responses in ECU were phase modulated in all four conditions, in both tied-belt conditions for TRI and in both split-belt conditions and Tied Fast for FCU. In the diagonal forelimb, responses occurred less frequently and consisted mostly of P2 responses during the period of activity. Diagonal ECU responses were phase modulated in all four conditions but not those in TRI.

**FIGURE 10:**
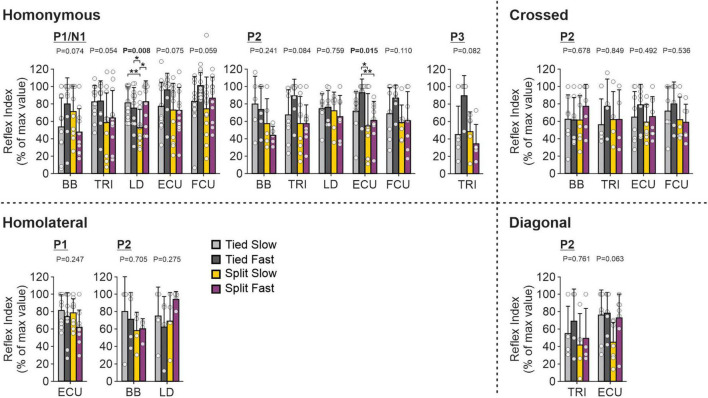
Forelimb reflex modulation across locomotor conditions for the group. Modulation indexes are shown for short- and longer-latency responses for all five forelimb muscles in the four locomotor conditions. Each bar represents the mean ± SD for pooled data. Note that only responses present in the four locomotor conditions in the same cat were pooled. *P*-values comparing conditions are indicated (main effect of repeated-measures ANOVA). Asterisks indicate significant differences between conditions (pairwise comparisons): *P < 0.05; **P < 0.01. BB, biceps brachii; TRI, triceps brachii; LD, latissimus dorsi; ECU, extensor carpi ulnaris; FCU, flexor carpi ulnaris.

To investigate and compare speed- and task-dependent modulation of cutaneous reflexes in forelimb muscles (Figure 10), we measured a modulation index (see section “2. Materials and methods”) for each locomotor condition by subtracting the smallest response from the largest response out of the 10 phases. Values were then normalized in each muscle for each response from the maximal value obtained in one of the four locomotor conditions. Only two homonymous muscles, LD and ECU, showed a significant task-dependent modulation. P1/N1 responses for LD were significantly smaller in the Split Slow condition compared to the other three conditions. For ECU, homonymous P2 responses were significantly smaller in both split-belt conditions compared to Tied Fast. For all crossed, homolateral or diagonal responses, we found no significant task-dependent differences.

### 3.4. Cutaneous reflex responses in hindlimb muscles

**FIGURE 11:**
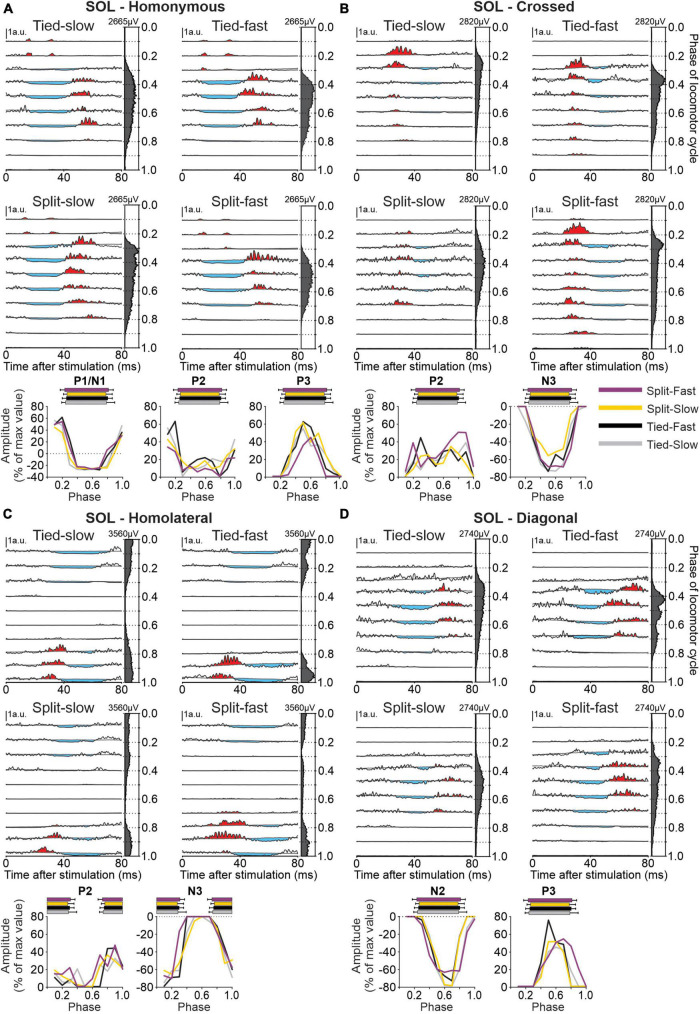
Intra- and interlimb reflexes in a hindlimb muscle during tied-belt and split-belt locomotion. Each panel shows reflex responses in 10 phases of the cycle evoked in the soleus (SOL) in a single cat by **(A)** stimulating the homonymous superficial peroneal (SP) nerve, **(B)** the crossed SP, **(C)** the homolateral superficial radial (SR) nerve, and **(D)** the diagonal SR in the four locomotor conditions. Black traces are averaged cycles that received a stimulation (*n* = 4–21 stimuli per phase) while gray traces are averaged cycles (blEMG) without stimulation (*n* = 74–137 cycles). Scale bars are shown in arbitrary units (a.u.) and are the same across phases and conditions for responses in each limb. Aligned vertically on the right side of each panel is the averaged rectified EMG of the SOL in the normalized cycle. At the bottom of each panel, the amplitude of short- and longer-latency responses is shown for the group within a normalized cycle to illustrate phase-dependent modulation. SP nerve stimulation evoked homonymous (*n* = 8 P1/N1 in 5/8 cats, *n* = 6 P2 in 5/8 cats and *n* = 8 P3 in 5/8 cats) and crossed (*n* = 6 P2 in 4/8 cats and *n* = 8 N3 in 6/7 cats) responses while SR nerve stimulation evoked homolateral (*n* = 6 P2 in 4/8 cats and *n* = 8 N3 in 5/8 cats) and diagonal (*n* = 7 N2 in 4/8 cats and *n* = 5 P3 in 3/8 cats) responses. Horizontal bars at the top represent the period of SOL activity for each locomotor condition (*n* = 7–19 control cycles) for pooled data.

To determine phase-, speed-, and task-dependent modulation of hindlimb cutaneous reflexes during quadrupedal locomotion, we recorded EMG responses in 10 muscles: SRT, ST, VL, IP, BFP, BFA, LG, SOL, MG, and TA. Figure 11 shows responses in SOL evoked by stimulating nerves in the homonymous SP, crossed SP, homolateral SR, and diagonal SR nerves in all four locomotor conditions. We selected the SOL, an ankle extensor, active during the stance phase because reflex responses from stimulating all four limbs were consistently evoked in this muscle. Stimulating the homonymous SP nerve (Figure 11A) evoked N1 responses that were followed by P3 responses when SOL was active as well as P1/P2 responses when inactive. P1/N1 and P3 responses were significantly modulated by phase in all four locomotor conditions for pooled data, while P2 responses were not. Crossed stimulation (Figure 11B) evoked P2 followed by N3 responses when SOL was active. N3 but not P2 responses were phase modulated. Homolateral SR nerve stimulation (Figure 11C) evoked P2 followed by N3 responses when the SOL was active, with no phase modulation for P2 responses except for the Tied Fast condition. Homolateral N3 responses were all significantly modulated by phase. Diagonal responses from the SR nerve (Figure 11D) consisted of N2 followed by P3 responses when SOL was active and both responses were phase modulated except in the Split Fast condition for P3 responses.

**FIGURE 12:**
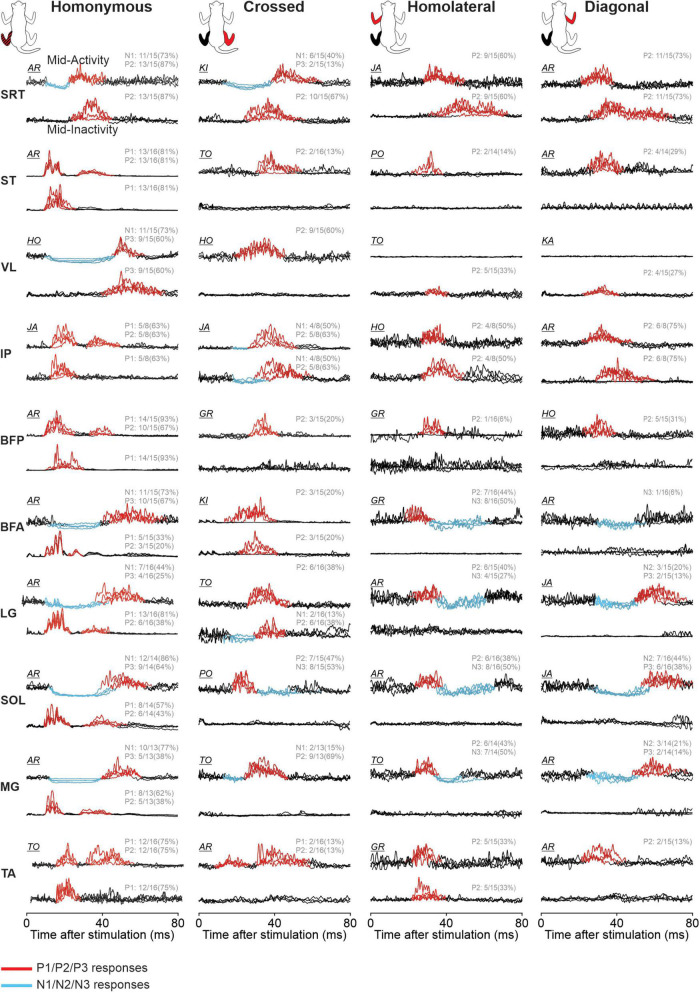
Phase-dependent modulation of hindlimb cutaneous reflexes during tied-belt and split-belt locomotion. Homonymous, crossed, homolateral, and diagonal responses are shown for anterior sartorius (SRT), semitendinosus (ST), vastus lateralis (VL), iliopsoas (IP), biceps femoris posterior (BFP), biceps femoris anterior (BFA), lateral gastrocnemius (LG), soleus (SOL), medial gastrocnemius (MG), and tibialis anterior (TA) muscles. The limbs stimulated and recorded in the cat diagram are displayed in red and black, respectively. Each black trace represents averaged stimulated cycles (*n* = 5–32 cycles) for a locomotor condition during a muscle’s period of mid-activity or mid-inactivity. The four locomotor conditions are superimposed for comparisons in a representative cat and optimized for display. Evoked responses are highlighted in red for positive and blue for negative. The fraction for each response indicates the proportion of pooled data evoking the same pattern of response in all locomotor conditions.

The response pattern evoked in the different muscles differed and representative examples are shown in Figure 12 for the 10 hindlimb muscles (BFA, BFP, IP, LG, MG, SRT, SOL, ST, TA, and VL) during their period of mid-activity and mid-inactivity. Table 4 summarizes the phase-dependent modulation of these responses for pooled data. We observed homonymous P1 or N1 responses in all muscles and significant phase modulation in all four conditions for SRT, ST, VL, LG, SOL, and MG. Homonymous P1/N1 responses did not show phase modulation for Split Slow in BFA and TA, as well as for Split fast in BFP and TA. Only IP had P1/N1 responses that were not phase modulated for all locomotor conditions. We observed homonymous P2 responses in all hindlimb muscles except in VL and BFA. Homonymous P2 responses were all phase modulated in ST and TA, excluding Split Slow and Split Fast, respectively. Those same responses only showed significant modulation for Tied Slow in SRT, for Tied Fast and Split Slow in BFP and for Tied Slow and Split Fast in LG. No significant phase modulation was found for homonymous P2 responses in IP, SOL and MG. Homonymous P3 responses were observed in VL, BFA, LG, SOL, and MG. SOL had homonymous P3 responses that were phase modulated in all four conditions but no significant modulation was found for other muscles of the triceps surae. Phase modulation in P3 responses was also present for VL and BFA in Split Fast and for both tied-belt conditions in BFA. In the crossed hindlimb, we observed P2 responses in six muscles, including LG, SOL, and MG that were not phase modulated in all locomotor conditions. In contrast, a significant phase modulation was observed for VL in Tied Fast and Split Slow, for IP in Tied Fast and Split Fast and SRT in Split Slow. Crossed N1 responses were observed in SRT and IP but were only phase modulated for all locomotor conditions in SRT. Only SOL had N3 responses, and they were all phase modulated. Homolateral hindlimb responses consisted exclusively of P2 and/or N3 responses. P2 responses were found in all muscles except in ST and BFP. Homolateral P2 responses in SRT were only phase modulated in both tied-belt conditions and Split Slow, in Tied Slow for LG and in Tied Fast for both SOL and MG. Homolateral N3 responses were found in BFA, LG, SOL, and MG and they were phase modulated for all locomotor conditions except LG only for Tied Fast. In the diagonal hindlimb, responses occurred less frequently and consisted mostly of P2 responses. We observed diagonal P2 responses with phase modulation in ST, VL, and BFP for Tied Slow, as well as in SRT and IP for Tied Fast. Finally, diagonal N2 and P3 responses were phase modulated for SOL in all four conditions except in Split Fast for P3 responses.

**TABLE 4:** Phase-dependent modulation of responses evoked by cutaneous inputs from the four limbs during tied-belt and split-belt locomotion in hindlimb muscles.

		Homonymous			Crossed			Homolateral		Diagonal		
		**P1/N1**	**P2**	**P3**	**P2**	**N1**	**N3**	**P2**	**N3**	**P2**	**P3**	**N2**
SRT	Tied slow	4.7 × 10^–6^	8.1 × 10^–3^		2.0 × 10^–1^	5.2 × 10^–4^		5.2 × 10^–4^		5.2 × 10^–2^		
	Tied fast	1.0 × 10^–10^	2.0 × 10^–1^		8.8 × 10^–2^	1.6 × 10^–3^		2.5 × 10^–3^		2.1 × 10^–2^		
	Split slow	2.9 × 10^–6^	3.4 × 10^–1^		4.6 × 10^–2^	3.6 × 10^–3^		4.7 × 10^–2^		9.4 × 10^–2^		
	Split fast	5.0 × 10^–6^	6.1 × 10^–2^		5.7 × 10^–2^	5.0 × 10^–5^		2.4 × 10^–1^		2.4 × 10^–1^		
ST	Tied slow	1.2 × 10^–6^	2.8 × 10^–6^							3.0 × 10^–2^		
	Tied fast	9.0 × 10^–6^	1.0 × 10^–5^							9.6 × 10^–2^		
	Split slow	4.1 × 10^–2^	6.6 × 10^–2^							8.0 × 10^–2^		
	Split fast	4.2 × 10^–4^	7.2 × 10^–5^							5.9 × 10^–2^		
VL	Tied slow	4.2 × 10^–4^		7.3 × 10^–2^	6.9 × 10^–2^			8.1 × 10^–2^		3.2 × 10^–2^		
	Tied fast	6.5 × 10^–5^		9.7 × 10^–2^	1.8 × 10^–2^			9.5 × 10^–2^		2.0 × 10^–1^		
	Split slow	5.2 × 10^–4^		1.1 × 10^–1^	1.5 × 10^–2^			9.1 × 10^–2^		7.0 × 10^–2^		
	Split fast	1.3 × 10^–2^		2.5 × 10^–2^	7.1 × 10^–2^			6.6 × 10^–2^		9.7 × 10^–2^		
IP	Tied slow	2.6 × 10^–1^	1.8 × 10^–1^		9.4 × 10^–2^	3.2 × 10^–1^		4.9 × 10^–1^		1.1 × 10^–1^		
	Tied fast	3.0 × 10^–1^	2.1 × 10^–1^		3.8 × 10^–3^	6.0 × 10^–2^		5.0 × 10^–1^		1.3 × 10^–2^		
	Split slow	1.9 × 10^–1^	1.6 × 10^–1^		7.2 × 10^–2^	1.0 × 10^–1^		7.2 × 10^–1^		2.3 × 10^–1^		
	Split fast	5.2 × 10^–1^	1.5 × 10^–1^		1.0 × 10^–2^	2.7 × 10^–1^		5.1 × 10^–1^		2.5 × 10^–1^		
BFP	Tied slow	4.5 × 10^–2^	2.2 × 10^–1^							3.5 × 10^–2^		
	Tied fast	3.6 × 10^–3^	1.9 × 10^–2^							8.8 × 10^–2^		
	Split slow	4.6 × 10^–2^	1.7 × 10^–2^							3.0 × 10^–1^		
	Split fast	8.2 × 10^–2^	2.6 × 10^–1^							8.4 × 10^–2^		
BFA	Tied slow	3.1 × 10^–2^		9.2 × 10^–4^				1.4 × 10^–1^	1.7 × 10^–5^			
	Tied fast	3.0 × 10^–3^		1.6 × 10^–2^				7.8 × 10^–2^	2.0 × 10^–3^			
	Split slow	6.1 × 10^–2^		1.7 × 10^–1^				1.9 × 10^–1^	7.2 × 10^–4^			
	Split fast	1.9 × 10^–2^		3.2 × 10^–3^				9.7 × 10^–2^	6.7 × 10^–5^			
LG	Tied slow	2.7 × 10^–7^	6.3 × 10^–2^	4.4 × 10^–1^	2.3 × 10^–1^			1.8 × 10^–2^	1.2 × 10^–1^			
	Tied fast	2.4 × 10^–5^	7.9 × 10^–2^	5.5 × 10^–1^	1.5 × 10^–1^			1.3 × 10^–1^	1.6 × 10^–2^			
	Split slow	2.5 × 10^–6^	3.3 × 10^–1^	4.4 × 10^–1^	2.2 × 10^–1^			2.9 × 10^–1^	5.4 × 10^–2^			
	Split fast	1.4 × 10^–4^	8.9 × 10^–3^	2.4 × 10^–1^	3.4 × 10^–1^			1.4 × 10^–1^	1.1 × 10^–1^			
SOL	Tied slow	1.2 × 10^–4^	1.6 × 10^–1^	3.2 × 10^–3^	3.7 × 10^–1^		8.1 × 10^–6^	2.7 × 10^–1^	9.5 × 10^–6^		3.3 × 10^–2^	1.4 × 10^–5^
	Tied fast	8.5 × 10^–5^	1.6 × 10^–1^	1.6 × 10^–3^	2.3 × 10^–1^		1.4 × 10^–3^	2.9 × 10^–2^	1.9 × 10^–6^		7.0 × 10^–3^	1.3 × 10^–5^
	Split slow	6.5 × 10^–5^	3.9 × 10^–1^	1.1 × 10^–3^	1.4 × 10^–1^		2.6 × 10^–3^	1.5 × 10^–1^	6.1 × 10^–5^		4.0 × 10^–2^	1.5 × 10^–5^
	Split fast	1.4 × 10^–4^	2.6 × 10^–1^	1.8 × 10^–3^	1.5 × 10^–1^		1.6 × 10^–4^	1.9 × 10^–1^	6.8 × 10^–5^		1.4 × 10^–1^	7.8 × 10^–4^
MG	Tied slow	4.0 × 10^–4^	4.5 × 10^–1^	1.5 × 10^–1^	2.5 × 10^–1^			5.6 × 10^–2^	2.7 × 10^–4^			
	Tied fast	4.5 × 10^–4^	2.0 × 10^–1^	2.6 × 10^–1^	7.2 × 10^–2^			3.4 × 10^–2^	3.5 × 10^–5^			
	Split slow	1.7 × 10^–2^	1.4 × 10^–1^	3.0 × 10^–1^	1.6 × 10^–1^			1.4 × 10^–1^	7.4 × 10^–4^			
	Split fast	9.4 × 10^–4^	5.0 × 10^–1^	2.8 × 10^–1^	2.2 × 10^–1^			6.8 × 10^–2^	2.4 × 10^–4^			
TA	Tied slow	1.5 × 10^–3^	4.2 × 10^–3^					3.2 × 10^–1^				
	Tied fast	7.1 × 10^–3^	1.9 × 10^–2^					1.9 × 10^–1^				
	Split slow	1.3 × 10^–1^	3.0 × 10^–3^					8.8 × 10^–2^				
	Split fast	1.5 × 10^–1^	1.9 × 10^–1^					1.8 × 10^–1^				

The table shows *P*-values (repeated-measures ANOVA) of phase-dependent modulation for all hindlimb reflex responses (P1, P2, P3, N1, N2, and N3) in the four locomotor conditions. Figure 12 provides details on the number of pooled data used each response. *P*-values in red indicate a significant phase-dependent modulation. SRT, anterior sartorius; ST, semitendinosus; VL, vastus lateralis; IP, iliopsoas; BFP, biceps femoris posterior; BFA, biceps femoris anterior; LG, lateral gastrocnemius; SOL, soleus; MG, medial gastrocnemius; TA, tibialis anterior.

**FIGURE 13:**
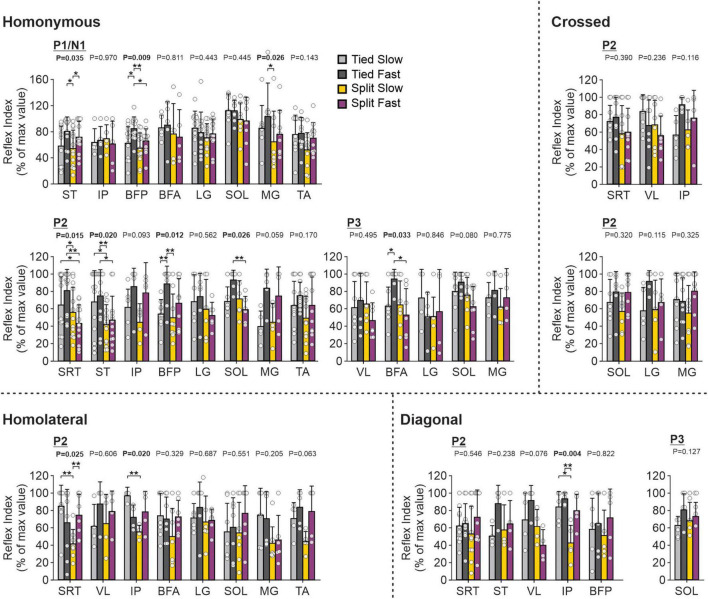
Hindlimb reflex modulation across locomotor conditions for the group. Modulation indexes are shown for short- and longer-latency responses for all ten forelimb muscles in the four locomotor conditions. Each bar represents the mean ± SD for pooled data. Note that only responses present in the four locomotor conditions in the same cat were pooled. *P*-values comparing conditions are indicated (main effect of repeated-measures ANOVA). Asterisks indicate significant differences between conditions (pairwise comparisons): *P < 0.05; **P < 0.01. SRT, anterior sartorius; ST, semitendinosus; VL, vastus lateralis; IP, iliopsoas; BFP, biceps femoris posterior; BFA, biceps femoris anterior; LG, lateral gastrocnemius; SOL, soleus; MG, medial gastrocnemius; TA, tibialis anterior.

To investigate and compare speed- and task-dependent modulation of hindlimb cutaneous reflexes (Figure 13), we measured modulation indexes. Comparisons between the different locomotor conditions (Tied Slow, Tied Fast, Split Slow, and Split Fast) were previously investigated in the intact and spinal cat during locomotion for three hindlimb muscles (VL, LG, and ST) but only with SP nerve stimulations (Hurteau and Frigon, 2018). They found that split-belt locomotion reduced cutaneous reflex modulation compared with tied-belt locomotion. In the present study, we extend these findings to other hindlimb muscles with SP and SR nerve stimulations. Most muscles were affected by condition depending on the response. When we found a significant main effect, pairwise comparisons revealed that it was mainly a decrease in reflex modulation between tied-belt and split-belt conditions. This was the case for homonymous P1 responses in ST (Tied Fast > Split Slow), BFP (Tied Fast > Split Slow and Split Fast) and MG (Tied Fast > Split Slow). This decrease was also observed for homonymous P2 responses in SRT (Tied Slow > Split Fast; Tied Fast > Split Slow and Split Fast), ST (Tied Slow > Split Slow; Tied Fast > Split Slow and Split Fast), BFP (Tied Fast > Split Slow); and SOL (Tied Fast > Split Fast), as well as for homonymous P3 responses in BFA (Tied Fast > Split Fast). For homolateral P2 responses, Split Slow showed smaller reflex modulation compared to Tied Slow in SRT and IP. For diagonal P2 responses in IP, Split Slow had smaller reflex modulation compared to both tied belt conditions. We also observed significant differences between tied-belt conditions or between split-belt conditions, with smaller values in Tied Slow and Split Slow compared to their fast counterpart. This was the case for homonymous P1 responses in ST (Split Fast > Split Slow) and BFP (Tied Fast > Tied Slow), for homonymous P2 responses in BFP (Tied Fast > Tied Slow), as well as for homonymous P3 responses in BFA (Tied Fast > Tied Slow). The same results were found for homolateral P2 responses in SRT between the two split-belt conditions (Split Fast > Split Slow).

## 4. Discussion

In the present study, we showed that cutaneous reflexes from forelimb and hindlimb cutaneous afferents are distributed to the four limbs during tied-belt and split-belt locomotion. The pattern of intra- and interlimb cutaneous reflexes and their phase-dependent modulation were conserved across tasks. However, short-latency cutaneous reflex responses to homonymous muscles were more likely to be evoked and phase modulated. We also confirmed similar spatiotemporal adjustments in the fore- and hindlimbs during tied-belt and split-belt locomotion at different speeds and slow-fast speed differences, respectively. Consistent with our hypothesis, when we did find significant difference in modulation of cutaneous reflexes in different tasks, it was mainly reduced during split-belt locomotion compared to tied-belt locomotion. In the following sections, we discuss the speed- and task-dependent modulation of the locomotor pattern and cutaneous reflexes, the functional significance of these responses during locomotion and possible modulatory mechanisms.

### 4.1. The forelimbs and hindlimbs display similar adjustments to speed and task

We observed similar spatiotemporal adjustments in the fore- and hindlimbs during tied-belt and split-belt locomotion (Figures 3, 4). For instance, during tied-belt locomotion, the stance phase and extensor burst durations shortened with increasing speed while step and stride lengths in the fore- and hindlimbs increased. During split-belt locomotion, stance and swing phase durations were longer and shorter on the slow side, respectively, in both the fore- and hindlimbs, in relation to the fast side where stance and swing were shorter and longer, respectively. Our findings are consistent with previous studies (Afelt et al., 1983; Blaszczyk and Dobrzecka, 1989; D’Angelo et al., 2014; Frigon et al., 2014; Thibaudier and Frigon, 2014; Kuczynski et al., 2017; Thibaudier et al., 2017; Lecomte et al., 2022). Similar adjustments are also observed in the hindlimbs of spinal cats (Forssberg et al., 1980; Frigon et al., 2013, 2017; Desrochers et al., 2019), indicating that they can be controlled at the spinal level. Rhythmic movements in the forelimbs/arms and hindlimbs/legs during human bipedal or animal quadrupedal locomotion are believed to be governed by similar spinal circuitry, such as central pattern generators (CPGs), at both cervical and lumbar levels (Zehr and Duysens, 2004; Zehr et al., 2004; Frigon, 2017).

### 4.2. Left-right coordination is more tightly regulated temporally than spatially

In the present study, we showed that left-right symmetry of the fore- and hindlimbs was more consistent on a step-by-step basis when considered temporally compared to spatially (Figure 7). The forelimbs and hindlimbs of cats display more consistent temporal and spatial left-right symmetry when stepping faster in the tied-belt condition, as shown previously (Frigon et al., 2014; Hurteau et al., 2017; Hurteau and Frigon, 2018). On the other hand, spatial left-right symmetry is considerably less consistent during split-belt locomotion compared to both tied-belt conditions, as previously shown (Hurteau and Frigon, 2018). The question is why left-right symmetry is more tightly regulated temporally as opposed to spatially, particularly during split-belt locomotion? The left-right symmetrical nature of walking is closely related to stability (Schöner et al., 1990; Collins and Stewart, 1993; Wilshin et al., 2017), referring to the body’s resistance against disruption of dynamic balance. For example, at low walking speeds, which is more asymmetric in terms of left-right coordination in cats and humans (Dambreville et al., 2015; Huijben et al., 2018), studies have found reduced stability and increased risk of falling in humans (Espy et al., 2010; Schniepp et al., 2012; Wuehr et al., 2014). Although there are definitely differences in stability between cat quadrupedal and human bipedal locomotion, studies have found similar changes in dynamic stability with increasing left-right speed differences in cats (Park et al., 2019; Latash et al., 2020) and humans (Buurke et al., 2019) during split-belt locomotion.

Dynamic balance control is closely associated with energy expenditure (Donelan et al., 2001), and in healthy humans, symmetric walking is believed to be energetically optimal (Srinivasan, 2011; Ellis et al., 2013). According to a simple model, the metabolic cost of leg movement is predicted to depend more heavily on step frequency compared to step length (Kuo, 2001), meaning greater changes in temporal features have a greater impact on energy cost compared to spatial ones. Split-belt locomotion experiments in humans have demonstrated that step time asymmetry plays a dominant role in shaping the energetic cost compared to step length asymmetry (Stenum and Choi, 2020). Other evidence in humans suggests distinct processes underlying temporal and spatial adaptations of walking for energy optimization, including the ability of step time asymmetry to reach equilibrium more quickly (Malone and Bastian, 2010; Malone et al., 2012) and to maintain it for longer periods (Darmohray et al., 2019; Gonzalez-Rubio et al., 2019) compared to step length asymmetry. Thus, there appears to be a need for stricter regulation of temporal left-right symmetry, which can be controlled at the level of spinal CPGs (McCrea and Rybak, 2008) and also by supraspinal inputs (Darmohray et al., 2019; Sato and Choi, 2019).

### 4.3. Short-latency reflex responses in homonymous muscles are more consistently evoked and more likely to be phase modulated

We found that homonymous short-latency excitatory (P1) and inhibitory (N1) responses were the most consistently evoked in forelimb and hindlimb muscles in all four locomotor conditions (Figures 9, 12). Homonymous P1 and N1 responses were also the most likely to be phase modulated in the four conditions (Tables 3, 4), consistent with previous results in our lab demonstrating preservation of short-latency cutaneous reflexes in hindlimb muscles and their phase-dependent modulation with changes in speed (Hurteau et al., 2017) and increased left-right asymmetry (Hurteau and Frigon, 2018) in intact and spinal cats. Homonymous longer-latency excitatory (P2) responses occurred less frequently and were less likely phase modulated, indicating that their modulation is not governed by the same mechanisms. We observed crossed, homolateral, and diagonal P2 responses in forelimb and hindlimb muscles less frequently and mainly during their period of activity. They were also less likely phase modulated. One exception was homolateral P1 and diagonal P2 responses in ECU that were phase modulated in all four locomotor conditions. We propose that homonymous are more likely to be phase modulated, reflecting more precise control, because homonymous pathways can rapidly correct limb trajectory whereas pathways to the other three limbs evoke responses to ensure that the animal maintains balance.

Different structures of the nervous system could be involved in generating and modulating short- and longer-latency responses. While P1/N1 responses are likely mediated exclusively by spinal pathways, longer-latency responses can involve additional synaptic contacts in the spinal cord and in supraspinal structures, as shown in cats (Fuwa et al., 1991; Frigon and Rossignol, 2008a; Frigon et al., 2009). Short-latency interlimb responses are mediated by circuits contained in the spinal cord because the minimal latency to evoke responses in hindlimb muscles through a pathway that traverses the brainstem, so-called spino-bulbo-spinal reflexes, is 18 ms in the cat (Shimamura and Livingston, 1963). Reflex responses are distributed to the four limbs via commissural neurons controlling left-right interactions at a segmental level and by short and long propriospinal neurons coupling spinal networks controlling the fore- and hindlimbs (Frigon, 2017). The decrease or loss of longer-latency reflexes in spinal-transected cats, including at high cervical levels, is consistent with a supraspinal contribution (Miller et al., 1977; LaBella et al., 1992; Hurteau et al., 2017; Hurteau and Frigon, 2018).

### 4.4. Functional considerations

Electrical or mechanical stimulation of the SP and SR nerves simulating contact of the paw dorsum evokes a stumbling corrective reaction during swing and a preventive reaction during stance. Phase-dependent modulation of cutaneous reflexes is crucial to ensure that the response pattern is functionally appropriate. The consistent phase-modulation of short-latency cutaneous reflexes during both tied-belt and split-belt locomotion suggests that this modulation is functionally important, because these responses are likely the ones that rapidly influence the locomotor pattern and correct stumbling reaction or prevent stumbling.

We showed that short-latency homonymous excitatory responses during the swing phase activate muscles that flex the knee (ST and BFP) and ankle (TA) in the hindlimbs and flex the elbow (BB) in the forelimbs, to rapidly move the limb away and over the obstacle during swing. Conversely, short-latency homonymous inhibitory responses during the stance phase reduce the activity of muscles that extend the hip (BFA), knee (VL), and ankle (MG, LG, and SOL) in the hindlimbs, and the elbow (TRI) and wrist (ECU) in the forelimbs, potentially to lower the center of gravity and prolong stance. During extensor and flexor activities, longer-latency excitatory responses (P2/P3) are frequently observed. These longer-latency responses allow the spinal circuits to correct limb trajectory and facilitate phase transitions. Longer-latency responses also allow integration of descending motor commands from the brain (Pruszynski and Scott, 2012). Supraspinal contributions are crucial for postural control and depend on the integration of somatosensory feedback that provides information on the body’s biomechanical state (Macpherson et al., 1997; Stapley et al., 2002; Frigon et al., 2021). Through sequential activation of forelimb and hindlimb muscles, reflex responses allow the perturbed limb to negotiate a simulated obstacle and maintain stability during forward progression.

Coordinating the four limbs during locomotion depends on complex interactions between different levels of the nervous system (reviewed in Frigon, 2017). Our study as well as others have shown that cutaneous inputs activate muscles in the four limbs during locomotion in healthy cats (Hurteau et al., 2018) and humans (Haridas and Zehr, 2003). These cutaneous reflexes can help the animal to maintain dynamic stability by regulating phase transitions and the duration of support periods. When the homonymous limb is perturbed during the swing phase, cutaneous reflexes can also reinforce limb stiffness of other limbs in their support phase by coactivating flexors and extensors. Coordinating the limbs is therefore critical for an effective locomotion and dynamic balance, whether it involves the left and right legs during human locomotion, and to a lesser extent the arms, or the four limbs during quadrupedal locomotion (Zehr et al., 2016; Frigon, 2017).

### 4.5. Left–right symmetry, stability, and reflex modulation

We showed that reflex modulation in homonymous hindlimb muscles was frequently reduced during split-belt locomotion compared to tied-belt conditions (Figure 13). A similar task-dependent modulation in homonymous forelimb muscles was found only for LD and ECU (Figure 10). The smaller number of significant task-dependent modulation of cutaneous reflexes in forelimb muscles could be because the forelimbs play a different role during quadrupedal locomotion, such as providing more body weight support or generating greater breaking forces compared to propulsive ones (Usherwood and Wilson, 2005). However, we sampled fewer forelimb muscles, which could also explain the smaller number of significant reflex modulation. The largest decrease in reflex modulation during split-belt locomotion was almost always observed when compared to the Tied Fast condition, which displayed the greatest left-right symmetry. For homolateral or diagonal responses in hindlimb muscles, when we observed weaker task-dependent reflex modulation, it was between one of the tied conditions and Split Slow. Thus, greater modulation of intra- and interlimb reflexes was associated with more consistent left-right symmetry, similar to our previous study (Hurteau and Frigon, 2018).

Studies have shown that cutaneous reflexes are increased in unstable locomotor conditions in humans (Haridas et al., 2005, 2006, 2008) but these studies did not measure left-right symmetry between the legs. We have previously proposed in intact and spinal cats that an increase in left-right asymmetry, particularly spatially, reduces walking stability, making it potentially less resistant to external perturbations (Hurteau et al., 2017; Hurteau and Frigon, 2018). Consequently, cutaneous reflex modulation is reduced in cats to avoid perturbing an unstable gait. Three-limb support, which provides stability, is reduced with increasing left-right speed differences during split-belt locomotion in intact cats (D’Angelo et al., 2014) and following a thoracic lateral hemisection in cats (Lecomte et al., 2022). The stability threats used in previous studies during human walking differ from the one potentially generated by split-belt locomotion. The difference in reflex modulation could also be due to species-dependent differences in limb/leg function (Usherwood and Wilson, 2005). In contrast to humans, cats heavily rely on their forelimbs for balance and support during locomotion. Our results are however similar to those found during beam walking where soleus H-reflexes were reduced (Llewellyn et al., 1990). Perhaps it can be argued that the stumbling corrective reaction has evolved to stabilize normal (tied-belt or symmetric) gait, but during unusual split-belt locomotion or in left-right asymmetric conditions, it has destabilizing effects and therefore is suppressed. What is clear is that reflexes, cutaneous and proprioceptive, are modulated by task for functional relevance.

### 4.6. Mechanisms involved in the modulation of cutaneous reflexes

Although we can only speculate, several mechanisms can be involved in modulation of cutaneous reflexes depending on speed and task. Because our cats were intact, all reflex responses can be controlled/modulated by spinal CPGs, supraspinal and propriospinal pathways as well as by afferent inputs entering the spinal cord. During walking, reflex responses are modulated relatively independent of background muscle activity when compared to static tasks (Duysens and Loeb, 1980; Buford and Smith, 1993; Van Wezel et al., 1997; Zehr et al., 1997; Haridas and Zehr, 2003; Hurteau et al., 2017, 2018; Hurteau and Frigon, 2018), indicating a premotoneural mechanism modulating transmission in reflex pathways. For example, presynaptic inhibition of cutaneous afferents and/or interneurons intercalated in the reflex pathway can reduce reflex strength (Burke, 1999; Rudomin and Schmidt, 1999). This presynaptic inhibition can be controlled by various sources, including central pattern generators, other spinal sensorimotor mechanisms, and supraspinal inputs (Menard et al., 2002; Bretzner and Drew, 2005; Sirois et al., 2013). Because we observed task-dependent modulation in spinal-transected cats (Hurteau et al., 2017; Hurteau and Frigon, 2018), a spinal mechanism is likely also involved in intact animals.

## 5. Conclusion

In conclusion, this study suggests common task-dependent mechanisms for regulating cutaneous reflex modulation in the fore- and hindlimbs. Similar phase-dependent modulation was observed in all four limbs under varying levels of left-right asymmetry. From a clinical perspective, individuals with locomotor deficits resulting from spinal cord injury or stroke often show gait asymmetry (Wilmut et al., 2017; Le Ray and Guayasamin, 2022) accompanied by changes in stretch and cutaneous reflexes (Frigon et al., 2009; Zehr and Loadman, 2012). While split-belt locomotion can improve gait in such individuals, it may not benefit all (Reisman et al., 2010). At present, we do not know how reflex responses in the four limbs are reorganized during locomotion and how these reflexes are modulated by task- and phase after neurological injury. We are currently investigating interlimb cutaneous reflexes during locomotion in cats before and after incomplete spinal cord injury under the same conditions.

## Data availability statement

The raw data supporting the conclusions of this article will be made available by the authors, without undue reservation.

## Ethics statement

The animal study was reviewed and approved by the Animal Care Committee of the Université de Sherbrooke.

## Author contributions

SM, IR, BP, and AF contributed to conception and design of the study. SM, CL, AM, JA, and JH conducted the research. SM organized the database and performed the data and statistical analysis. SM and AF wrote the first draft of the manuscript. All authors contributed to manuscript revision, read, and approved the submitted version.
